# Rural urbanisation and the effect on mental health

**DOI:** 10.1192/j.eurpsy.2023.1898

**Published:** 2023-07-19

**Authors:** C. Anghele

**Affiliations:** Psychiatry, “Prof. Dr. Al. Obregia” Hospital, Bucuresti, Romania

## Abstract

**Introduction:**

In the last 70 years there has been a massive change in rural versus urban distribution of the population (in the 1950’s only 30 % of the population had been living in urban areas, whereas in 2021 more than 55% were living in urban areas). This mass migration of the rural population, high density cities, traffic noise, severe pollution, high competition have made their mark on mental health, increasing the risk for various illnesses (schizophrenia). On the other hand, rural areas experience high rates of suicide, depression and a lack of access to the mental health workforce.

**Objectives:**

The goal of this research is to identify the effects of the rapid urbanization on the mental health in rural versus urban areas, as well as the impact of modernization in rural areas.

**Methods:**

For this we performed a literature search that synthesizes the newest research on the rural and urban mental health. Review type articles were excluded.

**Results:**

Results show a high frequency of schizophrenia, mood disorders or addictive disorders in urban areas and depression or alcohol dependence in rural areas. However, the improvement of the living conditions (such as Council of the Europe Development Bank), infrastructure, roads, water supply, bridges, sewerage networks have made their mark , modernizing rural economy. On the other hand existing barriers to mental health (desire to receive care, shortage of professionals in mental healthcare, lack of anonymity in treatment seeking, affordability or transportation to care, or even resources to learn) still remain to be addressed.

**Conclusions:**

The modernization of rural areas hasn’t changed the stigma for mental health. There is a need for increasing awareness on the impact of urbanization on mental health.

**Disclosure of Interest:**

None Declared

